# Non-Contact Face Temperature Measurement by Thermopile-Based Data Fusion

**DOI:** 10.3390/s23187680

**Published:** 2023-09-06

**Authors:** Faraz Bhatti, Grischan Engel, Joachim Hampel, Chaimae Khalil, Andreas Reber, Stefan Kray, Thomas Greiner

**Affiliations:** 1 Department of Engineering, Pforzheim University, 75175 Pforzheim, Germany; 2 Pyramid Computer GmbH, 79111 Freiburg, Germany

**Keywords:** non-contact temperature measurement, thermopile sensor, data fusion, intelligent access control system

## Abstract

Thermal imaging cameras and infrared (IR) temperature measurement devices act as state-of-the-art techniques for non-contact temperature determination of the skin surface. The former is cost-intensive in many cases for widespread application, and the latter requires manual alignment to the measuring point. Due to this background, this paper proposes a new method for automated, non-contact, and area-specific temperature measurement of the facial skin surface. It is based on the combined use of a low-cost thermopile sensor matrix and a 2D image sensor. The temperature values as well as the 2D image data are fused using a parametric affine transformation. Based on face recognition, this allows temperature values to be assigned to selected facial regions and used specifically to determine the skin surface temperature. The advantages of the proposed method are described. It is demonstrated by means of a participant study that the temperature absolute values, which are achieved without manual alignment in an automated manner, are comparable to a commercially available IR-based forehead thermometer.

## 1. Introduction

The non-contact measurement of skin temperature enables the early detection of potential signs of illness without the need for unwanted direct interaction with individuals. Additionally, it offers a practical solution for efficiently scanning large groups of people, supporting effective screening measures in both public and private spaces. Currently, there are two state-of-the-art non-contact methods:

- measurement of the forehead skin temperature using an infrared (IR) temperature measuring device;
- deployment of a thermal imaging camera at an exposed location for measuring the skin temperature.

Existing state-of-the-art approaches have certain limitations. The majority of these systems are based on IR temperature measurement sensors [1,2,3,4,5,6,7,8,9]. IR thermometer-based approaches do not allow for tracking the contour of the face. This requires the person’s face to be positioned within a predetermined frame, which can be error-prone and less convenient. As a result, they are unsuitable for deployment in crowded areas and are economically unviable due to the extensive need for personnel.

Systems based on IR thermal imaging [10,11,12] offer facial recognition and tracking, but they are significantly more expensive than conventional image sensors, rendering them economically impractical for many manufacturers of integrated systems. Current systems only provide temperature measurements based on the overall facial outline or non-specific facial regions. Specific facial areas are not considered or detected, making it impossible to determine temperature reliably and consistently in the same facial regions. This is crucial since facial skin temperature can vary significantly [13,14]. Furthermore, various capabilities for a fully automated solution are lacking. Some solutions have relay outputs which are indirectly controlled via Wi-Fi, requiring additional peripheral electronics. Current systems offer limited capabilities for remote reconfiguration, such as adjusting calibration data or individual measurement logic. Some researchers are combining RGB and thermal imagery for various applications, such as traffic monitoring and interdisciplinary inventory [15,16,17]. 

Only a few state-of-the-art approaches employ inexpensive thermopile sensors [18,19]. Thermopile-based systems currently lack facial recognition and/or tracking capabilities due to their limited resolution.

Given this context, the objective of this paper is to present an automated approach for contactless and facial area-specific skin temperature measurement. This method relies on the unique combination of an inexpensive thermopile sensor array and a 2D image sensor. Temperature and 2D image data are fused using a parametric affine transformation. A special calibration target is designed to determine this transformation. Through facial recognition, specific facial areas can be assigned with temperature values, which are then used to determine the skin surface temperature. Algorithms for detecting facial features and fusing data from the thermopile sensor array and 2D image sensor are described. Furthermore, the distributed system architecture and its components are introduced. Finally, the feasibility of the approach is demonstrated by a small participant study and the results are discussed.

## 2. Materials and Methods

### 2.1. Thermopile Sensor and Data Readout

For this study, a thermopile sensor with 60 × 40 pixels (HTPA60 × 40, from Heimann Sensor GmbH, Dresden, Germany) was chosen. Thermopiles are temperature sensors based on thermocouple elements consisting of two different conductor materials. One junction is opposed to the thermal radiation, generating a voltage signal proportional to the temperature difference to the other junction by the Seebeck effect [20]. 

Our sensor is controlled by a custom-programmed microcontroller. The integrated program involves reading calibration data from the sensor, capturing sensor raw data, and transmitting this data to a mobile PC via USB transfer. The calibration information is sensor-specific (e.g., sensitivity coefficients, number of defective pixels, etc.) and is required for the accurate calculation of the object temperature, as well as the configuration of the sensor’s clock frequency, ADC resolution, and the common mode voltage of the preamplifier. The calibration data are stored on an electrically erasable programmable read-only memory (EEPROM) in the sensor.

Since the object temperature calculation takes place on a mobile PC, the calibration data are transmitted once at the beginning of communication, while temperature raw data and other values (e.g., thermal drift) are continuously updated during processing. 

The mobile PC polls the sensor for raw data and corrects them based on the calibration information. The sensor raw data either provide a reference voltage proportional to the absolute temperature (PTAT) and the active pixels raw data or the electrical offsets, depending on the readout command. The ambient temperature is calculated from the sensor average measured PTAT value and from EEPROM calibration variables, such as the PTAT gradient and the PTAT offset. The sensor pixels voltages are subjected to different compensations before they can be used to determine the object’s temperature; initially, it is necessary to deduct the sensor’s thermal offset from each pixel to counteract potential thermal drift. Additionally, the outcome of the thermal gradient multiplied by the PTAT average is adjusted by the scaling coefficient for the thermal gradient stored in the EEPROM. Next, the electrical offsets are subtracted to compensate for changes in the supply voltage. Then a second supply voltage compensation (VddComp) is performed using the supply voltage of the sensor (Vdd) which is measured internally. After that, the sensitivity coefficients are calculated using EEPROM data. Finally, the sensitivity-compensated pixel values are calculated by dividing the pixels voltages by the sensitivity coefficients.

The sensitivity-compensated pixels and the ambient temperature are both needed to calculate absolute temperature for each pixel with the help of a look-up table, provided by the manufacturer. The look-up table rows represent the range of ambient temperatures supported by the sensor, and the columns represent the temperature values. When mapping the two values, a bilinear interpolation calculates the absolute object temperature for each pixel. As a result, temperature data matrices (thermopile images) of the captured scene are generated.

The measured temperature is also dependent on the emissivity [20]. Charlton et. al. have shown that the emissivity for human skin is nearly constant for all skin types of the Fitzpatrick scale [21]. Thus, in the following, the emissivity of skin is assumed to be constant with a value of ε=0.972, close to an ideal black body radiator. The result scales by $\sqrt[{4}]{\varepsilon }=\sqrt[{4}]{0.972}=0.993$ (see [20]), meaning it is only slightly influenced by the skin color. However, the measured skin temperature fluctuates due to changing ambient and physiological conditions.

### 2.2. Thermopile Sensor Characterization

**Figure 1:**
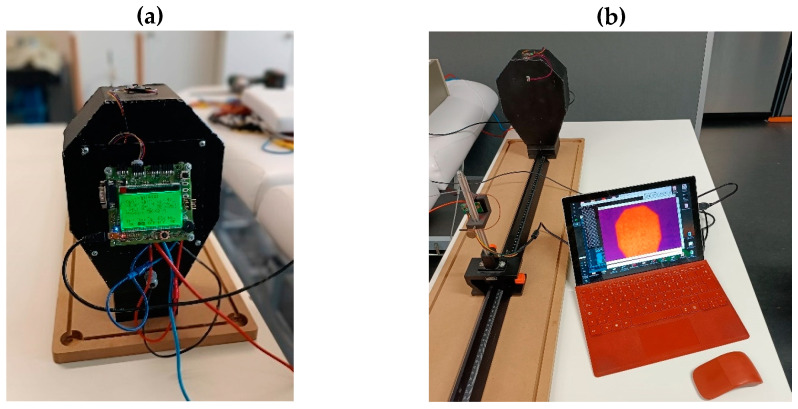
(**a**): backside of the artificial head showing the control electronics; (**b**): front side of the heated head, measured by the thermopile sensor. The artificial head provides a constant surface temperature and an emissivity similar to human skin.

An artificial head was built to characterize the sensor (see Figure 1). The head is constructed of sheet metal and painted with black paint to mimic a black body radiator with an emissivity close to 1. The head includes heating resistors inside at the bottom plate. External electronics allow the head to be set to a targeted temperature. The setup is used to characterize the sensor noise, the signal-to-noise ratio (SNR), and the frame rate. 

The pixel noise measured as mean squared error (MSE) using the *j*th sensor image $S_{j}\left(x,y\right)$ with discrete co-ordinates (x,y) of a homogeneous area with constant temperature μ, is ([22]):

(1) $$\mathrm{MSE}=\frac{1}{MN}\sum_{x=1}^{M}\sum_{y=1}^{N}{\left(S_{j}\left(x,y\right)-\mu \right)}^{2}$$

We extend this definition by taking a number of *J* temporal images into account:

(2) $$\mathrm{MSE}=\frac{1}{JMN}\sum_{j=1}^{J}\sum_{x=1}^{M}\sum_{y=1}^{N}{\left(S_{j}\left(x,y\right)-\mu \right)}^{2}$$

The root mean squared error (RMSE) is the square root of Equation (2). For a single pixel, the RMSE corresponds to the temporal standard deviation of that pixel.

The actual SNR is calculated using the signal (the constant temperature μ) and the MSE ([22]):

(3) $$\mathrm{SNR}=10\cdot \log_{10}\frac{\mu ²}{MSE}=20\cdot \log_{10}\frac{\mu }{RMSE}$$

For correlation analysis, we use the definition of the correlation coefficient r for two discrete lists of values, $p_{i}\;\mathrm{and}\;q_{i}$, and their respective averages, $\bar{p}$ and $\bar{q}$ [23]:

(4) $$r=\frac{\sum \left(\left(p_{i}-\bar{p}\right)\cdot \left(q_{i}-\bar{q}\right)\right)}{\sqrt{\sum {\left(p_{i}-\bar{p}\right)}^{2}\cdot \sum {\left(q_{i}-\bar{q}\right)}^{2}}}$$

### 2.3. Multimodal Sensor Setup and System Calibration

The imaging part of our system is comprised of the 60 × 40 thermopile sensor and a 2D color camera. The thermopile sensor array and the 2D image sensor are positioned closely together and are mechanically fixed. Although the camera is full-HD capable, we use a resolution of 600 × 400 in this study to have a better alignment to the thermopile resolution.

Both sensors image the same scene from a slightly different perspective. Consequently, calibration procedures used for stereo imaging might seem obvious. However, due to the completely different wavelength regions (~10 µm for the thermal sensor and 0.4 µm–0.7 µm for the visible range), commonly used methods fail. As the two sensors are based on different principles, have different resolutions, have different fields of view, and provide different types of data, feature-based algorithms cannot be applied. Intensity-based image approaches fail as the thermopile sensor provides temperature data and does not measure the scene’s visible light intensity.

**Figure 2:**
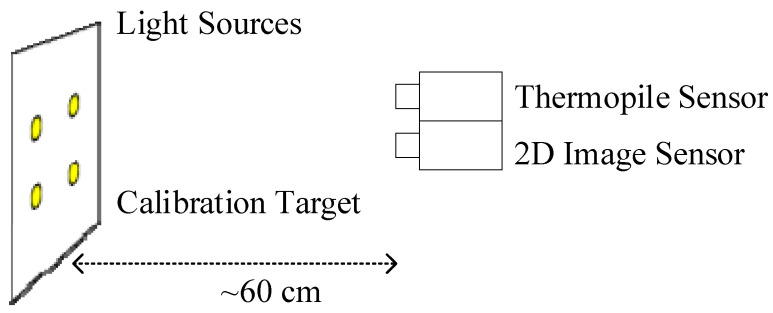
A thermopile sensor and a 2D color sensor image the calibration target.

We propose a modified calibration approach here. We identify correspondences between the data from the two sensors based on a contrast-rich calibration scene with distinct features. A custom calibration device is developed. It is used to generate circular features that exhibit significant temperature variations compared to the surroundings and emit light at the same time. This approach allows both sensors to capture these circular features with sufficient SNR for subsequent calibration algorithms. Figure 2 illustrates the sensor and calibration target.

**Figure 3:**
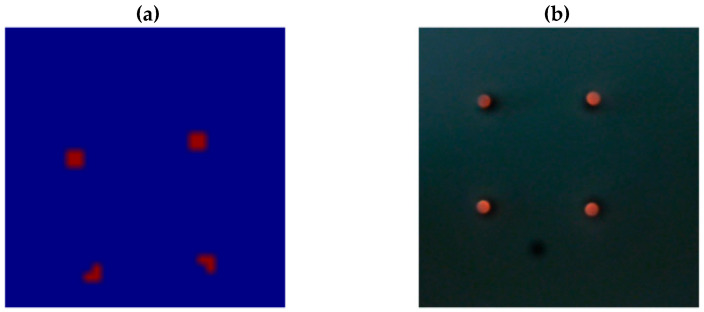
Image data with circular features, captured by the thermopile sensor (**a**), and by the 2D color image sensor (**b**).

The calibration target consists of a vertically oriented surface with four integrated, self-heated light sources. The two sensors are aligned to the calibration target at a distance of approximately 60 cm. Figure 3 illustrates the images captured by each respective sensor. The temperature data from the thermopile sensor are color coded.

The displayed circular features can be detected and matched to each other. The image from the 2D image sensor serves as reference, and corresponding circular features are searched in the image data from the thermopile sensor. Based on this information, an equation for a parametric affine transformation can be determined, taking into account scaling, rotation, shear, and translation of the data. In this manner we obtain a transformation, which allows us to match the thermopile image with the rgb image.

The steps of the algorithm for determining corresponding circular features and calculating a transformation matrix are in detail:

1. capturing the thermopile image *I_t_* and the camera image *I_c_*;
2. converting *I_t_* and *I_c_* into binary images to remove redundant information;
3. detecting the contours of the circular features in *I_t_* and *I_c_*;
4. determining the center points of the circular features, *I*_*t*0_, *I*_*t*1_, *I*_*t*2_, and *I*_*t*3_ in *I_t_*, as well as *I*_*c*0_, *I*_*c*1_, *I*_*c*2_, and *I*_*c*3_ in *I_c_*;
5. spatially sorting the detected center points from *I_t_* and *I_c_* to ensure correct correspondence;
6. calculating the real-valued coefficients *a*_0_, *a*_1_, *a*_2_, *b*_0_, *b*_1_, and *b*_2_ of the transformation matrix based on the centers of the circular features and the linear transformation equations:

$$x_{c}=a_{0}x_{t}+a_{1}y_{t}+a_{2}\;y_{c}=b_{0}x_{t}+b_{1}y_{t}+b_{2}$$

Result: transformation matrix $T=\left[\begin{array}{ccc} a_{0} & a_{1} & a_{2}\\ b_{0} & b_{1} & b_{2}\\ 0 & 0 & 1 \end{array}\right]$, which is used to transform the co-ordinates of a data point from the thermopile sensor into the co-ordinate system of the 2D image sensor.

The described algorithm is applied once before using the skin temperature measurement to determine the transformation matrix *T*. The calibration remains valid as long as the alignment between the two sensors is not altered. Thereafter, it can be employed within the sensor data fusion in combination with facial region detection and tracking, as described in the following section.

### 2.4. Skin Temperature Measurements and Signal Processing

The temperature of the facial skin can vary significantly in different areas, i.e., by more than 1 °C [13,14]. Therefore, for accurate determination of skin temperature, especially across different individuals, it is crucial to conduct targeted and consistent temperature measurements in specific facial regions.

Furthermore, during the measurement of a person’s temperature, it is essential to ensure that head movements do not distort the temperature measurement result. To detect facial contour points and specific facial regions, available state-of-the-art methods can be used [24]. Therefore, facial contour tracking is employed for dynamically adjusting the temperature determination. 

**Figure 4:**
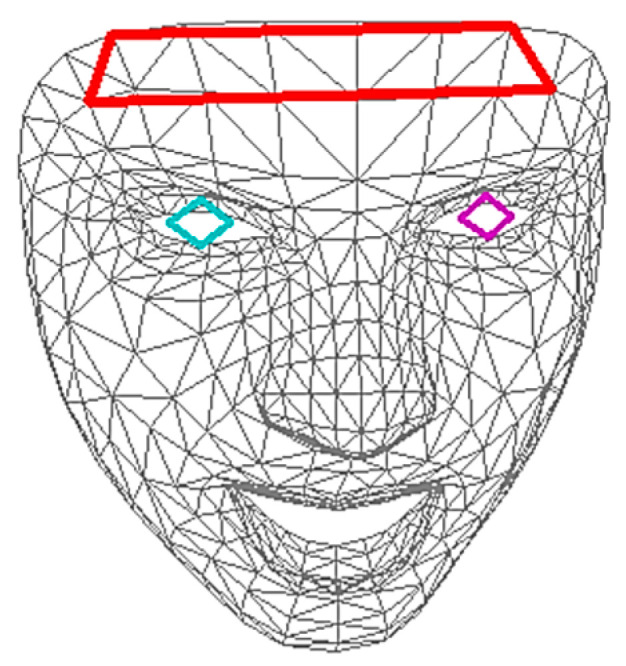
Mediapipe Face Mesh map of a person’s face detected by the color sensor. Each line in the image connects one of the 478 nodes. The eyes are marked by colored squares. The red quadrilateral marks the measured area on the forehead.

Figure 4 shows how this facial contour tracking is done. The face contour is detected with the help of Mediapipe Face Mesh, a machine learning framework provided by Google. Face Mesh determines characteristic landmarks within the face, making it possible to identify eyes, mouth, nose, and also forehead. The forehead region (red quadrilateral in Figure 4) is selected by using the proper Face Mesh nodes (node numbers 68, 103, 297, and 333 are used in this work). The red quadrilateral covers an area of approximately of 8 cm × 1.5 cm = 12 cm^2^, measured at a working distance of 50 cm.

With the help of the transformation matrix T, the thermopile image is transformed to match the rgb image. All mapped thermopile temperature values within the forehead region are averaged spatially. The landmarks are continuously tracked, even during slight face movements. Thus, it is possible to identify the same area within consecutive frames. These areas are then also averaged temporally over a time interval (e.g., 1 s, 5 s), depending on the settings of the software. All steps are performed in real time, which allows for continuous detection and tracking of the corresponding region. Ultimately, temperature data for the entire facial area are always available during the measurement process and can be evaluated accordingly. 

The steps of the algorithm for combined sensor data fusion with facial region detection and tracking are, in detail:

1. capturing the thermopile image *I_t_* and the camera image *I_c_*;
2. determining the transformed image *I_t’_* from *I_t_*. For each point *(x_t_* and *y_t_)* in *I_t_*, the following transformation equation applies:
  $$\left[\begin{array}{c} x^{’}\\ y^{’}\\ 1 \end{array}\right]=T\left[\begin{array}{c} x_{t}\\ y_{t}\\ 1 \end{array}\right]$$
  The transformation matrix *T* is obtained from the initial sensor calibration shown above; *x*’ and *y*’ represent the transformed points in *I_t’_*;
3. identifying the forehead landmark points G_c_ based on *I_c_* (using Face Mesh);
4. estimating the temperature data within the area enclosed by G_c_ using *I_t’_*;
5. spatially averaging the temperature data from *I_t’_* within the respective area;
6. temporally averaging the temperature data from *I_t’_* within the respective area;
7. visualization of the fused image with corresponding facial features in *I_c_*.

### 2.5. Distributed System Architecture and Components

This section presents the system architecture used. It is designed to be fundamentally re con figurable, enabling flexible adaptation to different requirements of various ap pli ca tion scenarios. The corresponding system architecture consists of five fundamental components:

- mobile PC with an integrated touchscreen [25];
- thermopile sensor: Heimann HTPA60 × 40d sensor with ARM Cortex M0 (Pyramid Computer GmbH, Freiburg, Germany);
- binocular camera with two lenses: 2MP AI dual lens camera module (1920 × 1080, RGB and IR camera, Hampo Electronic Technology, Dongguan, China);
- electronic relays which can be connected to further actuators;
- RFID reader;
- edge server system for providing configuration parameters.

In this context, the mobile PC plays a central role. Besides providing an interactive display to visualize the measurement process, it takes charge of the entire measurement and evaluation logic. The thermopile sensor with an integrated microcontroller is connected to the mobile PC via a USB serial interface. The processing of raw sensor data is conducted in real time on the mobile PC, using the Python programming language, along with the software frameworks OpenCV (v 4.6.0) and Google Mediapipe (v 0.8.2). The acquisition of the raw sensor data from the thermopile sensor on the ARM Cortex-M0 is accomplished using the C programming language. Additionally, the system architecture can be optionally incorporated into a broader cloud/edge system. This enables remote and location-independent adjustments of both (sensor-specific) configuration parameters and individual measurement logic. Furthermore, the mobile PC features an RFID reader and universal switching outputs (relays) which can be utilized to control automated processes such as actuators based on temperature measurements for access control tasks.

## 3. Results

### 3.1. Sensor Characteristics

The sensor was characterized by imaging the artificial head, which was heated to a constant temperature of ~33 °C. Figure 4 shows the mean and standard values of the thermopile sensor over a time interval of 5 s.

**Figure 5:**
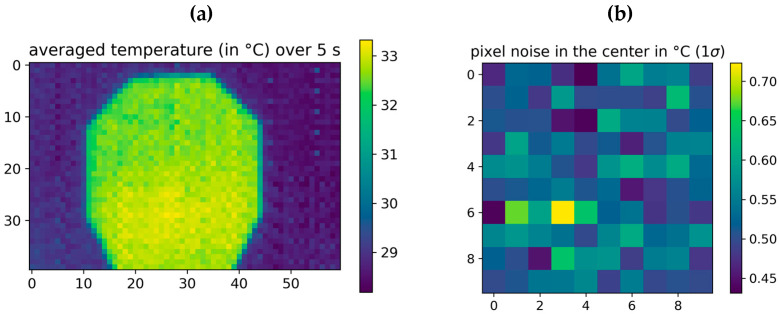
Averaged temperature image (**a**) and temporal standard deviation of 10 × 10 pixels in the upper homogeneous region of the image (**b**). Both images were taken over a time interval of 5 s. The frame rate of the thermopile sensor is 22 frames per second. The exposure time of the sensor is not controllable by us, it is set internally in the sensor.

The averaged temperature values in Figure 5a are showing a fixed pattern noise imposed on the image. The temperature values of the head slightly increase towards the lower end where the heating resistors are located. The pixels in the upper, more homogeneous part show a pixel noise between σ=0.45 °C−0.7 °C (Figure 5b), on average approximately σ=0.53 °C, which is quite high for most applications.

**Figure 6:**
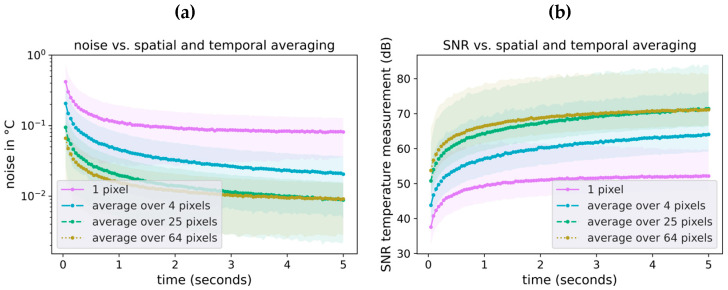
Effect of spatial and temporal averaging on noise (**a**) and SNR (**b**). The noise and SNR values are obtained and calculated from the measurement of the artificial head shown in Section 2.2 with a constant surface temperature μ. Noise values are calculated as RMSE for pixel areas of different sizes. Temporal averaging is done by averaging successive frames over a given time span. SNR values are calculated according to Equation (3).

Pixel noise, determined according to Equation (2), can be improved by spatial and temporal averaging, shown in Figure 6. The noise can be decreased by spatial averaging, assuming a homogeneous surface area is measured. The noise is reduced by 50% by averaging 4 pixels (see Figure 6). By averaging a few dozen pixels, the statistical noise is dramatically reduced, also eliminating variations due to fixed pattern noise. A further noise reduction is possible by additional temporal averaging. The statistical temperature noise reaches levels of σ=0.01 °C by averaging over 64 pixels for 5 s. The SNR of a single temperature pixel is 36.6 dB. Averaging 64 pixels over 5 s increases the SNR value to ~70 dB. In this manner, spatial and temporal averaging allows for precise temperature measurements even with noisy thermopile sensors.

### 3.2. System Characteristics

**Figure 7:**
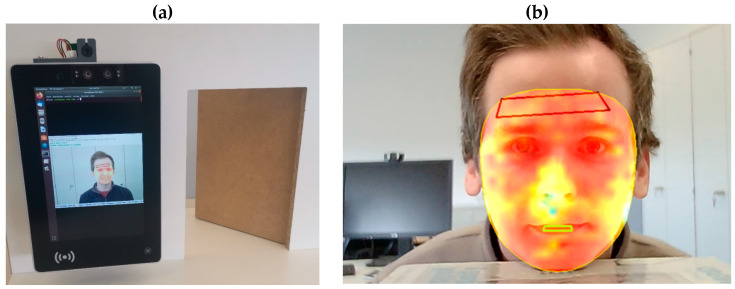
(**a**): System prototype; (**b**): exemplary illustration of the combined sensor data fusion with facial region detection and tracking.

Figure 7a illustrates the overall implemented system. The mobile PC is equipped with an integrated camera and the thermopile sensor is added on top. The system prototype can be connected via an electronic relay to an actuated door, which served as a use-case scenario for temperature-based access control.

The Figure 7b shows the detection of both the facial contour and specific facial regions, such as the forehead. The face is tracked continuously. The skin surface temperature is visualized through a colormap. The red quadrilateral shows the area used for determining forehead temperature. The region is identified with the help of Face Mesh and mapped to the thermopile image, consisting of roughly 25 thermopile pixels. The pixels are spatially averaged to one temperature value. Furthermore, the area is tracked during several seconds (5 s, in this case) and averaged over this time interval to the final temperature value. The system is capable of performing all necessary calculations in real time. 

Inside and outside temperature as well as physical activities influence the measured skin temperature. We performed some qualitative measurements and found that the ambient temperature has a correlation coefficient of r≈0.35 (calculated by Equation (4)). The distance to the subject also influences the result and has a correlation coefficient of r≈0.27. However, these influences are difficult to reproduce. 

**Figure 8:**
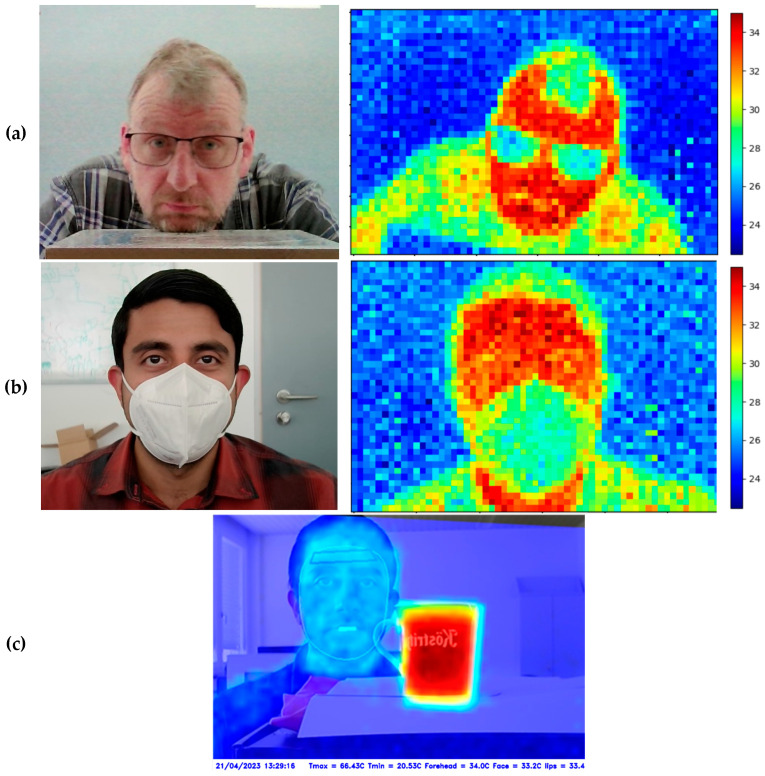
Temperature measurements and identification of foreign objects, such as glasses, mask, or hot objects. (**a**,**b**): Each image pair shows the rgb image and the corresponding thermopile image before registration. (**c**): The image shows the fused rgb and thermopile image after registration.

Foreign objects located near the face or covering specific facial areas can adversely affect the measurement of skin temperature. Examples of such objects include wearing a mask or glasses. The proposed approach enables the indirect detection of these objects by assuming that skin areas covered by a foreign object have lower temperatures than uncovered areas. In this way, foreign objects can be identified by detecting cooler temperature regions on the face. The described principle is exemplified in Figure 8.

To detect specific foreign objects, certain facial regions (e.g., chin, mouth, nose, and cheek areas, corresponding to the position of a mask) can be defined. Each facial region corresponds to a set of temperature values. To identify a foreign object in a facial region, the number of temperature values is counted which deviate from the expected temperature range. If the number is too high, the person is asked to remove any objects present in the facial region. Otherwise, temperature values below the defined threshold are filtered out and are not considered in the temperature measurement. 

Even larger objects do not disturb the temperature measurement. The last example of Figure 8 shows that despite the presence of a hot cup with a temperature of approximately 66 °C, the face is recognized, and the surface temperature of the forehead (34 °C) is accurately determined.

### 3.3. Participant Study and Quantitative Measurement Results 

To validate the temperature measurement approach, especially regarding the surface forehead temperature, a participant study was conducted. The system was calibrated as described in Section 2.3. The calibrated system was validated by holding a hot object (e.g., a hand or a cup) at different points in the image and ensuring that both image sources matched in the fused image. The temperature data were compared to those produced by a commercially available, manually operated forehead thermometer (Medisana TM A79). The study involved five participants with skin types I, II, and V according to the Fitzpatrick scale. Five measurements were conducted for each individual under room temperature conditions (22 °C). For better repeatability, all measurements were conducted without any physical activities of the participants. All subjects had approximately the same distance of 50 cm–60 cm to the sensor. The participants remained immobile to provide more stable results. Approximately 50 images from the thermopile sensor were captured and subsequently spatially as well as temporally averaged over a five-second period. The reference measurements with the forehead thermometer were performed manually. 

For each participant, the time, reference temperature as well as thermopile temperature were measured. The five participants were measured one after each other, and the procedure was repeated five times. The raw data of the thermopile system were corrected by the emissivity factor of 0.99, as mentioned in Section 2.1. The average values and the standard deviations have been calculated for each participant, as shown in Table 1. The standard deviation fluctuates approximately between 0.07 °C and 0.24 °C for the thermopile and reference measurement. The mean 1σ deviation of the reference measurement is $\sigma_{ref}=0.14\;{}^{\circ}C\;\left(2\sigma_{ref}=0.28\;{}^{\circ}C\right)$ and $\sigma_{tp}=0.12\;{}^{\circ}C\;\left(2\sigma_{tp}=0.24\;{}^{\circ}C\right)$ for the thermopile sensor. 

**Figure 9:**
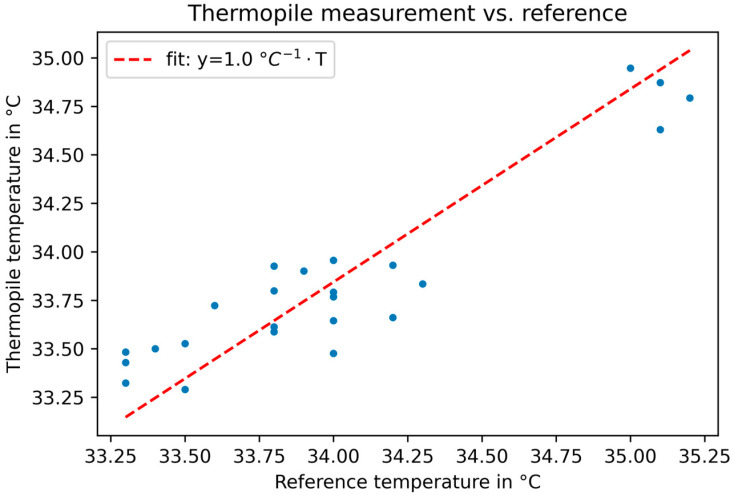
Measurements of the forehead temperature with the Medisana TM reference device compared to our thermopile sensor. A linear correlation between the measurements was found with a fitted slope of 1.0. The emissivity of skin was assumed to be ε=0.972.

The results of the measurements are presented in Figure 9, illustrating a linear correlation with a slope of approximately 1 (after correction for skin emissivity) between the data from the commercially available forehead thermometer and the temperature data by our setup. The correlation coefficient between the reference and thermopile measurements is calculated to be r=0.92, indicating a very strong correlation between those values. The RMSE (compare Equation (2)) between the corrected dataset and reference measurement is calculated to be RMSE = 0.22 °C, indicating that the measured absolute values are comparable to those of the commercial reference system.

## 4. Discussion 

Our thermopile sensor has a native pixel noise of approximately 0.53 °C. This noise value is higher than most manually operated temperature devices. However, through spatial and temporal averaging, the noise value can be brought into a subordinate range of <<0.1 °C. Averaging also increases the SNR accordingly.

While the ratio between thermopile measurements and reference measurements closely approximates 1 in our scenario, we recommend conducting an initial validation of this factor. A low-cost generic thermometer produced results that differed slightly from those obtained with the Medisana reference thermometer. According to our findings, a sample size of 3–5 individuals is sufficient to check the quality and the scaling factor of the thermometer.

In the participant study, a temperature noise of σ = 0.12 °C was measured on average for the individual participants. The temperature noise observed in this study is higher than statistically anticipated. This means, that this noise is probably caused by systematic deviations due to, e.g., fluctuations in skin temperature, blood perfusion, varying environmental conditions, or movements of the person which are not corrected properly by the face tracking. We did not observe relevant differences in the temperature measurements for different skin phototypes according to the Fitzpatrick scale. This finding is also supported by other studies [21].

In our study, we mostly used controlled conditions. For example, the distance was not varied, the subjects were staying at room temperature, and did not move excessively. Prior to the participant study, we qualitatively investigated skin temperature changes. We found that temperature slightly increases over distance and is also dependent on ambient temperature conditions, e.g., coming from the cold outside. This problem affects not only our measurement methodology, but all non-contact methods based on thermal imaging. 

Our system can be used, for example, for contactless temperature measurements in combination with an access control system. It can be connected directly to further actuators, such as electronic doors, via an electronic relay interface. We were able to implement such a temperature-based access control connected to a door as a use case scenario in the lab. The covid pandemic has shown that such systems are needed at the entrance to critical areas, like airports, hospitals, nursing homes, or large buildings, such as residential complexes. 

However, further studies are necessary to determine the temperature under more difficult conditions, such as wearing reflective glasses, face masks, headpieces, scarfs or under changing ambient conditions. Moreover, the accuracy of the measurement can decrease as the 2D image sensor and thermopile sensor move farther away from the face. This is because the captured forehead area in the 2D image covers a smaller area with fewer pixels due to the increased distance. As a consequence, spatial and temporal averaging might yield less accurate results. A higher spatial resolution of the thermopile sensor could potentially reduce this effect and enable better registration results. Additionally, further noise suppressing techniques, such as bandpass filtering, 2D image filters, etc., might be helpful to reduce noise. Furthermore, our system features a second, independent infrared camera, which could be beneficial for operating the system under low light conditions.

Additional work is also needed to understand external influences to skin temperature. This is a challenging task as it is necessary to understand and monitor more parameters in the system. A further aspect might be to include heart rate monitoring [26], remote photoplethysmography [26], or blood perfusion detection [27]. Integrating these parameters into the analysis, along with temperature measurements from extremities like arms, hands, and toes, could lead to a holistic understanding of the body’s response to different external stimuli. This knowledge might be used to infer core body temperature, which is an important vital sign and used commonly for diagnosing fever. Ultimately, leveraging inverse heat transfer methods [28,29,30] in conjunction with these physiological indicators could significantly enhance the prediction of the thermal state of the human subject. Inverse heat transfer relies on simulation models, working backward from temperature measurements to the boundary conditions or heat sources that could have caused those measurements. This might offer insights into inferring the core body temperature accurately.

## 5. Conclusions

This paper presents an approach for automated, contactless, and region-specific measurement of skin surface temperature on the face. The method is based on data fusion from a thermopile sensor and a 2D image sensor. By capturing and tracking the facial outline, specific temperature values are assigned to selected facial areas, which are used to determine the skin surface temperature accurately.

The application of the proposed approach was demonstrated. In qualitative terms, the participation study has shown that facial capture and tracking reduce the susceptibility to errors in temperature measurement, particularly when foreign objects are in close proximity to the face. In quantitative terms, the subject study demonstrated that the measured temperature absolute values have an RMSE of 0.22 °C, rendering them comparable to those of a commercially available, manually operated reference system. As such, our system holds the potential to become a valuable tool in the future for accurate and automated non-contact temperature measurements.

## Figures and Tables

**Table 1:** Average and standard deviation for all study participants. For each person, five measurements were performed and used for calculating mean and standard deviation (1σ).

Participants	${µ}_{ref}\;$(°C)	$\sigma_{ref}$ (°C)	${µ}_{tp}$ (°C)	$\sigma_{tp}$ (°C)
1	34.06	0.174	33.64	0.116
2	33.9	0.237	33.71	0.242
3	35.1	0.071	34.81	0.116
4	33.82	0.133	33.83	0.074
5	33.36	0.08	33.45	0.07

## Data Availability

The data are not publicly available due to confidentiality rules.
